# Antioxidant, Anti-Tyrosinase and Anti-Inflammatory Activities of Oil Production Residues from *Camellia tenuifloria*

**DOI:** 10.3390/ijms161226184

**Published:** 2015-12-10

**Authors:** Shu-Yuan Chiou, Choi-Lan Ha, Pei-Shan Wu, Chiu-Ling Yeh, Ying-Shan Su, Man-Po Li, Ming-Jiuan Wu

**Affiliations:** 1 Crop Environment Section, Hualien District Agricultural Research and Extension Station, Hualien 973, Taiwan; sychiou@mail.hdais.gov.tw; 2 Department of Health and Nutrition, Chia-Nan University of Pharmacy and Science, Tainan 717, Taiwan; choilanha@mail.cnu.edu.tw (C.-L.H.); susan_fanny_sun@yahoo.com.tw (Y.-S.S.); 3 Department of Biotechnology, Chia-Nan University of Pharmacy and Science, Tainan 717, Taiwan; dc7575@gmail.com (P.-S.W.); goat933318u@gmail.com (C.-L.Y.); jeremy810407@gmail.com (M.-P.L.)

**Keywords:** *Camellia tenuifloria*, monophenolase, diphenolase, iNOS, macrophages

## Abstract

*Camellia tenuifloria* is an indigenous *Camellia* species used for the production of camellia oil in Taiwan. This study investigated for the first time the potential antioxidant, anti-tyrosinase and anti-inflammatory activities of oil production byproducts, specifically those of the fruit shell, seed shell, and seed pomace from *C. tenuifloria*. It was found that the crude ethanol extract of the seed shell had the strongest DPPH scavenging and mushroom tyrosinase inhibitory activities, followed by the fruit shell, while seed pomace was the weakest. The IC_50_ values of crude extracts and fractions on monophenolase were smaller than diphenolase. The phenolic-rich methanol fraction of seed shell (SM) reduced nitric oxide (NO) production, and inducible nitric oxide synthase (iNOS) expression in lipopolysaccharide (LPS)-stimulated RAW 264.7 cells. It also repressed the expression of IL-1β, and secretion of prostaglandin E_2_ (PGE_2_) and IL-6 in response to LPS. SM strongly stimulated heme oxygenase 1 (HO-1) expression and addition of zinc protoporphyrin (ZnPP), a HO-1 competitive inhibitor, reversed the inhibition of NO production, indicating the involvement of HO-1 in its anti-inflammatory activity. The effects observed in this study provide evidence for the reuse of residues from *C. tenuifloria* in the food additive, medicine and cosmetic industries.

## 1. Introduction

The genus *Camellia*, which includes about 100–250 species of East Asian evergreen shrubs and trees, belongs to the tea family (Theaceae) [1,2]. It is an important economic crop with various applications. The leaves of *C. sinensis* are processed to create tea, while the seeds of *C. oleifera*, *C. tenuifloria,* *C. japonica*, and to a smaller amount of other species, such as *C. crapnelliana*, *C. reticulata*, *C. sasanqua,* *C. grijsii*, and *C. sinensis*, are pressed to produce camellia oil. Camellia oil has a high content of monounsaturated fatty acid, oleic acid, and is extensively used in culinary, medical, and cosmetic practices in East Asia [3].

*Camellia tenuifloria* is an indigenous *Camellia* species native to Taiwan [4]. Along with *C. oleifera*, they are the two main *Camellia* for the production of camellia oil in Taiwan [5]. The fruit shell, seed shell, and seed pomace of *Camellia* are byproducts of oil production and are always discarded or used as fertilizer. However, bioactive phytochemicals, such as saponins, flavonoid glycosides, and polysaccharides, are reported in the seed pomace or shell of *C. oleifera*, in addition to triglycerides [6,7,8,9,10,11], and anti-microbial, antioxidant, anti-inflammatory, and analgesic effects have also been disclosed for these substances [7,8,9,10,11,12,13,14]. In contrast, there is only one report regarding the antioxidant and anti-aging activities of *C. tenuifolia* seed pomace [15], while the other potential biological properties of the residues of *C. tenuifolia* remain to be further examined. 

Reactive oxidative species (ROS) are constantly generated in the body from internal metabolism and external exposure. Oxidative stress is an imbalance between the production of ROS and the ability of the body to counteract or detoxify their harmful effects [16]. Oxidative stress has been implicated in cardiovascular, cancer, neurodegenerative, diabetes, aging, and other age-dependent diseases [17]. Many phytochemicals in fruit, vegetables, grains, and other plant foods have been linked to reductions in the risk of these diseases [18,19,20], either by directly scavenging ROS, or by modulating cell signaling pathways [21].

Tyrosinase (EC 1.14.18.1) is a ubiquitous enzyme found in nearly all cells. It has a binuclear copper center and catalyzes two different reactions. Monophenolase is responsible for the orthohydroxylation of monophenols (such as tyrosine) to *o*-diphenols (such as l-DOPA, 3-(3,4-dihydroxyphenyl)-l-alanine); while diphenolase catalyzes the oxidation of *o*-diphenols (such as l-DOPA) to *o*-quinones (such as dopaquinone) [22]. Dopaquinone in turn can be readily converted to dopachrome, an orange to red pigment. Unfavorable enzymatic browning of plant-derived foods by tyrosinase can cause a decrease in nutritional quality and the formation of toxic compounds [23]. Tyrosinase also plays a critical role in the biosynthesis of melanin in melanocytes and is considered to be the key enzyme in coloring of the skin, hair, and eyes [24]. Inhibition of tyrosinase is, thus, one of the major strategies that the cosmetic industry uses to achieve skin-whitening effects and depigmentation after sunburn [25]. Kojic acid is the most intensively studied tyrosinase inhibitor widely used as a cosmetic bleaching agent and as a food additive for preventing discoloration. It shows a competitive inhibitory effect on monophenolase activity and a mixed inhibitory effect on the diphenolase activity of mushroom tyrosinase [26]. Among the plant extracts, flavonoids are the main tyrosinase inhibitor constituents [23]. The inhibitory mechanism of flavonol inhibitors is mainly competitive inhibition for monophenolase and copper chelation [26].

Inflammatory responses are typically present as a series of vascular and cellular reactions initiated by injury or infection. Macrophages play a critical role in the initiation, maintenance, and resolution of inflammation. They secrete pro-inflammatory cytokines [27] and inflammatory mediators, such as prostaglandins (PGs) and nitric oxide (NO), to augment the host’s defenses against invasion by microbes [28,29,30]. Sustained and chronic inflammation may underlie the pathogenesis of arthritis, cancer, stroke, as well as neurodegenerative and cardiovascular diseases [30].

To better understand the potential usages of residues from oil production of *Camellia tenuifloria*, we analyzed the antioxidant, anti-tyrosinase, and anti-inflammatory activities of the crude ethanol extracts and different partition fractions of the fruit shell, seed shell, and seed pomace. The fractions with the strongest anti-nitric oxide production activity were further used to study the molecular mechanism underlying its anti-inflammatory activity. 

## 2. Results

### 2.1. Antioxidant Activities of Crude Ethanol Extracts and Different Partition Fractions of Fruit Shell, Seed Shell, and Seed Pomace of C. tenuifloria 

DPPH (2,2-Diphenyl-1-(2,4,6-trinitrophenyl)hydrazyl) scavenging activity and the total phenolic contents of crude ethanol extracts and different fractions, namely *n*-hexane, methanol, *n*-butanol and aqueous fractions, from the fruit shell, seed shell, and seed pomace of *C. tenuifloria* were determined as described in Materials and Methods. Table 1 shows that crude ethanol extracts of fruit shell (FE) and seed shell (SE) exhibited stronger DPPH scavenging activity than that of seed pomace (PE). As for the partition fractions, the methanol fractions derived from crude extracts of fruit shell, seed shell and seed pomace (FM, SM, and PM) showed the strongest antioxidant activities as compared with other fractions, and their IC_50_ were 7.34 ± 0.89, 5.47 ± 0.28, and 14.38 ± 0.23 μg/mL, respectively. Methanol fractions had the highest phenolic contents, followed by *n*-butanol fractions; while aqueous fractions had the lowest. Surprisingly, we found that the aqueous fraction of the seed shell (SA) contained only a modest level of phenolic content but had strong DPPH scavenging activity. This indicates that SA might contain water-soluble non-phenolic antioxidant components. There was no detectable DPPH scavenging activity or phenolic content for the *n*-hexane factions.

**Table 1:** Antioxidant activities of crude extracts and different fractions of fruit shell, seed shell, and seed pomace of *C. tenuifloria*.

Samples	IC_50_ for DPPH Scavenging (μg/mL) ^a^	Total Phenolic Content (mg GAE/g dw) ^c^
Fruit Shell		
Crude ethanol extract (FE)	19.74 ± 0.19	107.37 ± 3.54
*n-*Hexane fraction (FH)	ND ^b^	ND ^b^
Methanol fraction (FM)	7.34 ± 0.89	266.79 ± 1.85
*n-*Butanol fraction (FB)	13.18 ± 0.75	129.13 ± 2.55
Aqueous fraction (FA)	27.25 ± 1.30	51.85 ± 3.16
Seed Shell		
Crude ethanol extract (SE)	14.30 ± 1.01	91.42 ± 1.47
*n-*Hexane fraction (SH)	ND ^b^	ND ^b^
Methanol fraction (SM)	5.47 ± 0.28	266.30 ± 7.29
*n-*Butanol fraction (SB)	15.55 ± 0.10	106.95 ± 3.09
Aqueous fraction (SA)	5.82 ± 0.09	88.90 ± 5.71
Seed Pomace		
Crude ethanol extract (PE)	84.96 ± 2.75	62.40 ± 3.26
*n-*Hexane fraction (PH)	ND ^b^	ND ^b^
Methanol fraction (PM)	14.38 ± 0.23	120.56 ± 2.16
*n-*Butanol fraction (PB)	170.99 ± 16.69	43.34 ± 0.27
Aqueous fraction (PA)	218.03 ± 23.12	6.26 ± 1.6
α-Tocopherol (control)	11.89 ± 1.14	

^a^ IC_50_ for DPPH scavenging : concentration (in μg/mL) necessary for reduction 50% DPPH radical. Data are represented as the mean ± SD (*n* = 3); ^b^ ND: not detectable; ^c^ mg GAE/g dw: minigrams of gallic acid equivalent per gram of dry weight. Data are represented as the mean ± SD (*n* = 3).

### 2.2. Anti-Tyrosinase Activities of Crude Ethanol Extracts and Different Partition Fractions of Fruit Shell, Seed Shell, and Seed Pomace of C. tenuifloria

**Figure 1:**
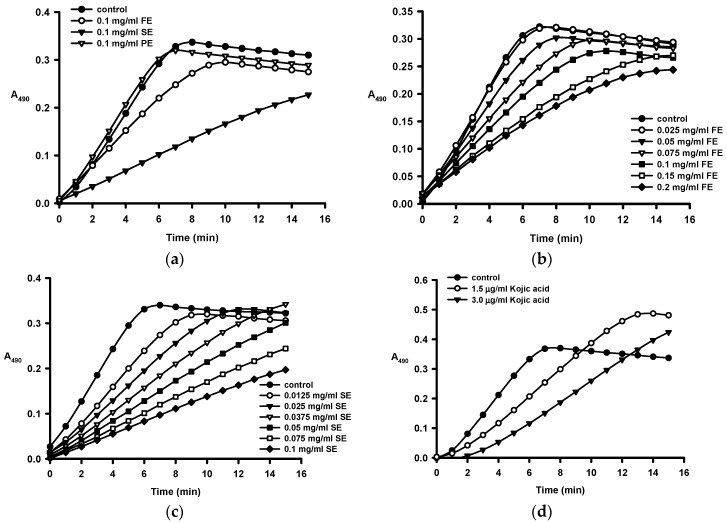
Progress curves for the inhibition of monophenolase activity of tyrosinase (**a**) by the crude ethanol extracts of the fruit shell (FE), seed shell (SE), and seed pomace (PE) of *C. tenuifloria*; (**b**) by various concentrations of the crude ethanol extracts of fruit shell (FE); (**c**) by various concentrations of the crude ethanol extracts of seed shell (SE); and (**d**) by kojic acid. Monophenolase activity was analyzed by monitoring the change in the OD at 490nm (A_490_) as a function of time as described in the Materials and Methods.

We then investigated the existence of tyrosinase inhibitors in the biowaste of *C. tenuifloria* by testing 0.1 mg/mL of ethanol extracts of fruit shell (FE), seed shell (SE), and seed pomace (PE) against monophenolase activity of mushroom (*Agaricus bisporus*) tyrosinase because its commercial availability and well-studied biochemical kinetic characterization [23,31]. Figure 1a shows that in the absence of an inhibitor tyrosinase-catalyzed dopachrome production reached a plateau after 8 min due to substrate depletion. In the presence of FE, no new dopachrome was formed after 10 min, while continuous dopachrome was produced during the test period (15 min) when SE was present. It was found that SE inhibited tyrosinase activity most potently, followed by FE, while PE did not have any detectable inhibitory effect by comparing their initial velocities. 

Figure 1b,c further demonstrate that the inhibition of monophenolase increased with increasing concentrations of FE and SE. Figure 1d shows that kojic acid, a well-studied tyrosinase inhibitor, exhibited a stronger inhibitory activity against monophenolase than FE and SE, and a lag phase was noted at 3 μg/mL. This result indicates that monophenolase was strongly inhibited and required time to produce sufficient amount of DOPA for the subsequent diphenolase reaction [26].

**Figure 2:**
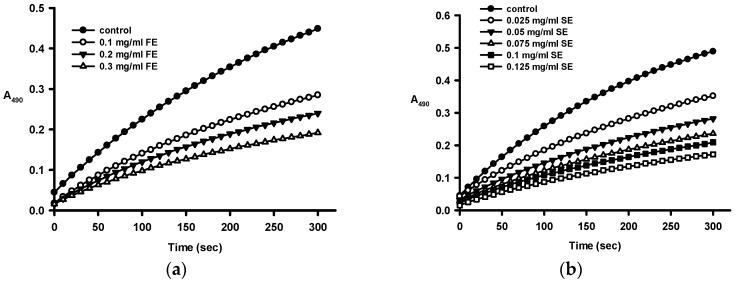

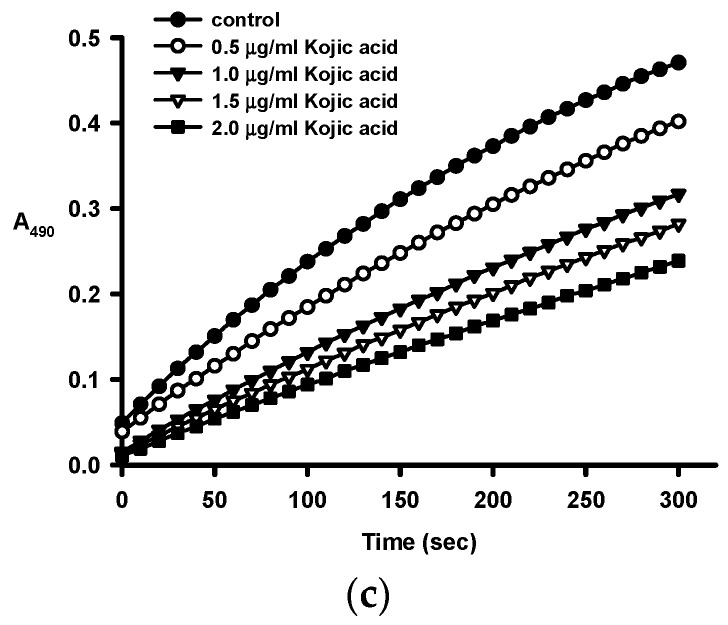
Progress curves for the inhibition of diphenolase activity of tyrosinase (**a**) by various concentrations of the crude ethanol extracts of fruit shell (FE); (**b**) by various concentrations of the crude ethanol extracts of seed shell (SE); (**c**) by kojic acid. Diphenolase activity was analyzed by monitoring the change in A_490_ as a function of time as described in the Materials and Methods.

We also investigated the effect of crude ethanol extracts of fruit shell (FE) and seed shell (SE) on diphenolase activity by using l-DOPA as a substrate. Figure 2 shows that both FE and SE inhibited the diphenolase activity dose-dependently.

The anti-tyrosinase potencies of partition fractions from fruit shell and seed shell extracts were further investigated, and their IC_50_ values are shown in Table 2. The *n*-butanol fraction (FB) showed the strongest inhibitory activity against monophenolase and diphenolase as compared with other fractions of fruit shell. For the fractions of seed shell, the anti-monophenolase potency was in the order of SB ≈ SA > SM, while the anti-diphenolase was SB >SA ≈ SM; indicating that various kinds of anti-tyrosinase components might exist in seed shell. No detectable anti-tyrosinase activity could be found in the *n*-hexane fractions.

**Table 2:** Anti-tyrosinase activities of crude extracts and different fractions of fruit shell and seed shell of *C. tenuifloria.*

Samples	IC_50_ for Monophenolase (μg/mL) ^a^	IC_50_ for Diphenolase (μg/mL) ^a^
Fruit Shell		
Ethanol crude extract (FE)	112.1 ± 2.2	307 ± 9
*n-*Hexane fraction (FH)	ND ^b^	ND ^b^
Methanol fraction (FM)	539 ± 13	256 ± 18
*n-*Butanol fraction (FB)	70.0 ± 5.5	219 ± 12
Aqueous fraction (FA)	276.7 ± 9.1	675 ± 26
Seed Shell		
Ethanol crude extract (SE)	36.1 ± 0. 2	58.9 ± 2.5
*n-*Hexane fraction (SH)	ND ^b^	ND ^b^
Methanol fraction (SM)	61.5 ± 0.3	94.6 ± 3.0
*n-*Butanol fraction (SB)	32.7 ± 0.4	62.8 ± 0.9
Aqueous fraction (SA)	33.7 ± 0.2	93.9 ± 3.5
Kojic acid (control)	2.81 ± 0.01	1.63 ± 0.09

^a^ IC_50_ for tyrosinase : concentration (in μg/mL) necessary for inhibition of 50% activity. Values are represented as mean ± SD (*n* = 3); ^b^ ND: not detectable.

### 2.3. Anti-Inflammatory Activities of Residues from C. tenuifloria

#### 2.3.1. Effects of Crude Ethanol Extracts and Different Partition Fractions of Fruit Shell, Seed Shell, and Seed Pomace of *C. tenuifloria* on LPS-Induced Nitric Oxide (NO) Production and Cytotoxicity in RAW 264.7 Cells

**Figure 3:**
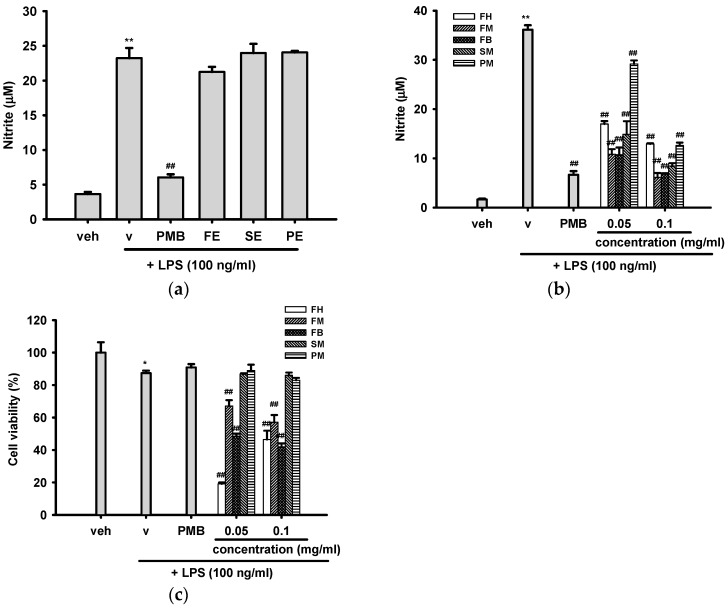
Effects of crude ethanol extracts and different partition fractions of fruit shell, seed shell, and seed pomace of *C. tenuifloria* on LPS-induced nitrite oxide (NO) production and cytotoxicity in RAW 264.7 cells. (**a**) RAW 264.7 macrophages were cultured with indicated reagent at 37 °C for 18 h in a 96-well plate. The nitrite production was determined by the Griess reaction (**b**,**c**) RAW 264.7 macrophages were cultured with indicated reagent at 37 °C for 24 h in a 96-well plate. The nitrite production was determined by the Griess reaction, and the cell viability was analyzed by MTT assay. Data are represented as the mean ± SD (*n* = 3). * *p* < 0.05; ** *p* < 0.01 represents significant differences compared with the vehicle control (without LPS); ^##^ *p* < 0.01 represents significant differences compared with the LPS-treated vehicle.

We first investigated whether the crude ethanol extracts of fruit shell (FE), seed shell (SE), and seed pomace (PE) of *C. tenuifloria* can function as inhibitors for NO release. Figure 3a shows that stimulation of cells with LPS (100 ng/mL) in the presence of vehicle (0.1% ethanol) for 18 h induced a significant increase in nitrite production from the basal level 3.65 ± 0.29 to 23.23 ± 1.47 μM. Co-treatment of cells with LPS and polymyxin B (PMB, 10 μg/mL), an endotoxin neutralizing peptide, inhibited nitrite production to 6.04 ± 0.44 μM. On the other hand, none of tested ethanol extracts (0.1 mg/mL) had detectable NO inhibitory activity. 

We continued to screen which of the partition fractions exhibited NO inhibitory effects. Figure 3b shows that stimulation of cells with LPS (100 ng/mL) in the presence of vehicle (0.1% DMSO) for 24 h induced a significant increase in nitrite production from the basal level 3.67 ± 0.15 to 36.15 ± 0.89 μM. Positive control, PMB (10 μg/mL) inhibited LPS-induced NO production to 6.67 ± 0.74 μM. Co-treatment of cells with LPS and 0.05 or 0.1 mg/mL of hexane (FH), methane (FM), and *n*-butanol (FB) fractions of fruit shell, as well as methanol fractions of seed shell (SM) and seed pomace (PM), could significantly inhibit NO production dose-dependently. The strongest inhibitory effects were observed for FM and FB. There was no detectable anti-nitric oxide activity for the rest of the partition fractions.

LPS induces apoptosis in macrophages mostly through the production of pro-inflammatory mediators [32]. A decrease in the cell viability of the LPS group as compared with the vehicle control indicates the cytotoxic effect of NO (Figure 3c). Cell viability after 24 h of SM or PM (0.05 and 0.1 mg/mL) co-treatment was more than 95% of that seen with the LPS group, implying that the diminished NO production by SM or PM was not due to cell death. In comparison, FH, FM, and FB (0.05 and 0.1 mg/mL) significantly enhanced LPS-induced cytotoxicity, indicating their inhibition against NO production is likely due to cytotoxic effects.

#### 2.3.2. Effects of Methanol Fractions of Seed Shell (SM) and Seed Pomace (PM) on LPS-Mediated Inducible Nitric Oxide Synthase (iNOS) Expression 

**Figure 4:**
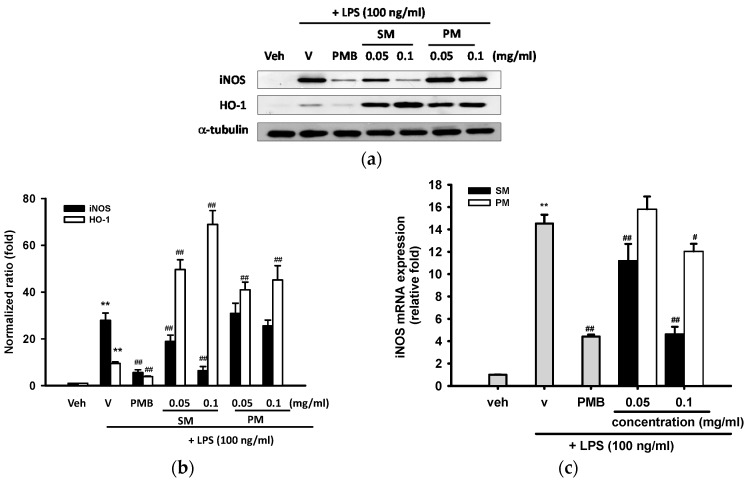
Effects of methanol fractions of seed shell (SM) and seed pomace (PM) on LPS-mediated iNOS and HO-1 expression. (**a**) RAW 264.7 macrophages were cultured with indicated reagent in 6-well plates for 16 h. Total cell lysates were prepared and the iNOS and HO-1 protein expression were detected by Western blotting, as described in Materials and Methods. The levels of α-tubulin in the total lysates serve as the loading control; (**b**) Band intensities were quantified by ImageJ software and indicated as relative folds of iNOS/α-tubulin and HO-1/α-tubulin. This experiment was replicated three times with similar results; (**c**) RAW 264.7 cells were cultured as described above for 12 h. Total RNA was prepared and the mRNA levels of iNOS were quantified by RT-Q-PCR relative to β-actin, as described in Materials and Methods. Data are represented as the mean ± SD (*n* = 3). ** *p* < 0.01 represents significant differences compared with the vehicle control (without LPS); ^#^ *p* < 0.05; ^##^ *p* < 0.01 represent significant differences compared with the LPS-treated vehicle.

Western blot analysis was carried out on whole cell lysates of RAW 264.7 cells in order to determine whether SM or PM exerted NO inhibition on activated macrophages by blocking iNOS expression. α-tubulin serves as a loading control to make sure equal amounts of protein were analyzed. Figure 4a,b shows that RAW 264.7 macrophages expressed a minimal level of iNOS protein expression, and LPS (100 ng/mL) caused a ~28-fold increase after 16 h treatment. PMB (10 μg/mL) significantly decreased LPS-induced iNOS protein expression by ~80%. In comparison, SM (0.05 and 0.1 mg/mL) reduced LPS-upregulated iNOS protein expression by 32% and 78%, respectively. However, PM did not significantly attenuate LPS-mediated iNOS protein expression, although NO production was partially inhibited (Figure 3b).

Reverse transcription real-time PCR (RT-Q-PCR) was used to further investigate the effects of SM and PM on iNOS mRNA expression. RAW 264.7 cells treated with LPS (100 ng/mL) for 12 h caused a 14.5-fold increase, as compared with the vehicle control group, and the addition of PMB (10 μg/mL) blocked LPS-mediated iNOS mRNA expression by 70% (Figure 4c). SM (0.05 and 0.1 mg/mL), in conjunction with the stimuli, inhibited iNOS mRNA induction significantly and dose-dependently by 23% and 68%, respectively (*p* < 0.01). Surprising, we found that PM (0.1 mg/mL) slightly decreased iNOS expression by 17% (*p* < 0.05), although no change on iNOS protein could be found. This discrepancy may be due to a difference in the detection threshold between RT-Q-PCR and Western blot analysis.

#### 2.3.3. Effects of Methanol Fractions of Seed Shell (SM) and Seed Pomace (PM) on LPS-Mediated Heme Oxygenase 1 (HO-1) Expression

Heme oxygenase 1 (HO-1) has emerged as an important antioxidant and anti-inflammatory enzyme [33]. Many researches demonstrate that HO-1 induction prevents inflammatory responses in macrophages [34,35,36]. Figure 4a,b show that treatment of RAW 264.7 cells with LPS (100 ng/mL) in the presence of vehicle (0.1% DMSO) for 16 h induced a 9.5-fold increase in HO-1 protein expression. The addition of PMB (10 μg/mL) significantly decreased LPS-induced HO-1 protein expression by ~60% (*p* < 0.01). On the other hand, 0.05 and 0.1 mg/mL SM enhanced LPS-stimulated HO-1 protein expression by 5.2- and 7.2-fold, respectively, as compared with the level seen in the LPS group. PM also exerted significant increases in HO-1 protein expression (*p* < 0.01), although it had much weaker inhibitory effect against iNOS induction.

**Figure 5:**
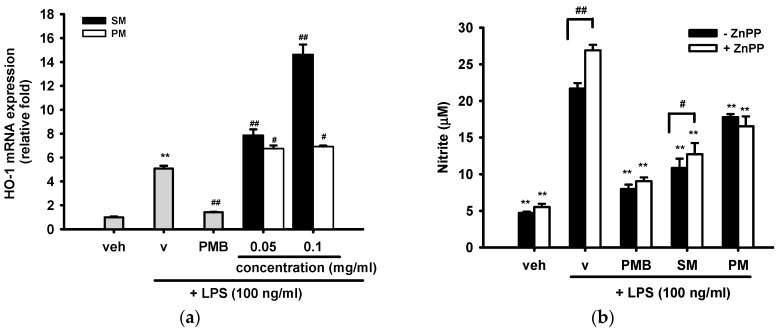
Involvement of HO-1 up-regulation in the anti-inflammatory effect of the methanol fraction of seed shell (SM). (**a**) RAW 264.7 macrophages were cultured with indicated reagent in 6-well plates for 12 h. Total RNA was prepared and the mRNA levels of HO-1 were quantified by RT-Q-PCR relative to β-actin. Data are represented as the mean ± SD (*n* = 3). ** *p*< 0.01 represents significant differences compared with the vehicle control (without LPS). ^#^ *p* < 0.05; ^##^ *p* < 0.01 represent significant differences compared with the LPS-treated vehicle; (**b**) RAW 264.7 cells were treated with indicated reagent in the presence or absence of ZnPP (10 μM) for 18 h, and nitric oxide produced in the medium was determined by the Griess reaction. Data are represented as the mean ± SD (*n* = 3). ** *p* < 0.01 indicates significant differences from the respective LPS-treated group; ^#^ *p* < 0.05; ^##^ *p* < 0.01 represent significant differences from ZnPP untreated group.

Figure 5a shows that treatment of cells with LPS (100 ng/mL) for 12 h caused a 5.1-fold increase in HO-1 mRNA expression, as compared with the vehicle control group. SM and PM co-treatments exerted additive effects on HO-1 expression. The induction effect of SM (*p* < 0.01) was dose-dependent and was much stronger than that of PM (*p* < 0.05). On the other hand, PMB (10 μg/mL) significantly blocked LPS-induced HO-1 mRNA expression by 72% (*p* < 0.01).

We further investigated whether the anti-inflammatory effects of SM and PM on nitric oxide production was mediated through HO-1. Figure 5b shows that zinc protoporphyrin IX (ZnPP, 10 μM), a potent competitive inhibitor of HO enzyme activity, significantly enhanced LPS-mediated NO production (*p* < 0.01) and slightly reversed the inhibition of NO by SM (*p* < 0.05). This result indicates that HO-1 activity participated in part in the anti-inflammatory mechanism of SM. However, no effect was found for the PMB or PM groups.

#### 2.3.4. The Inhibitory Effects of Methanol Fractions of Seed Shell (SM) and Seed Pomace (PM) on LPS-induced Prostaglandin E_2_ (PGE_2_) Release and Expression of Cyclooxygenase-2 (COX-2) 

**Figure 6:**
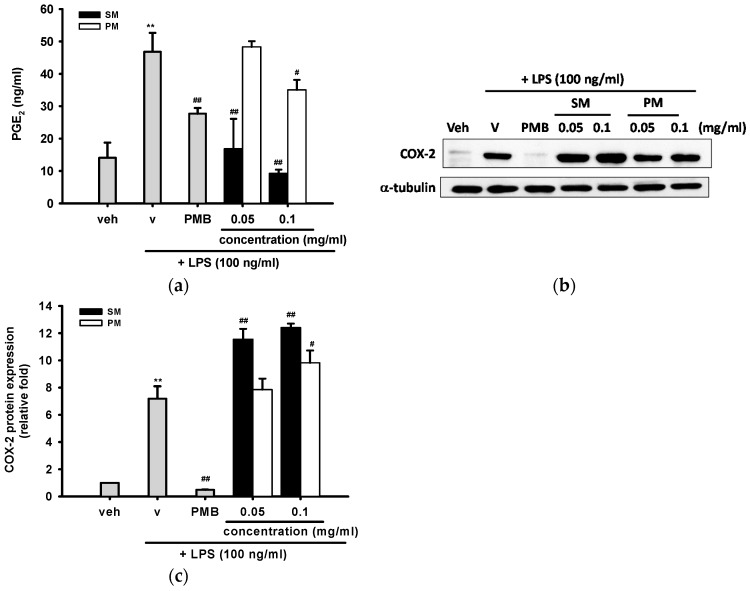
The inhibitory effects of methanol fractions of seed shell (SM) and seed pomace (PM) on LPS-induced PGE_2_ release and protein expression of COX-2. (**a**) RAW 264.7 cells were treated with indicated reagent in 96-well pleats for 18 h and the culture media were collected for PGE_2_ assay as described in Materials and Methods; (**b**) RAW 264.7 macrophages were cultured with indicated reagent in six-well plates for 16 h. Total cell lysates were prepared and the COX-2 protein expression were detected by Western blotting, as described in Materials and Methods; (**c**) The levels of α-tubulin in the total lysates serve as the loading control. Band intensities were quantified by ImageJ software and were indicated as a relative fold of COX-2/α-tubulin. This experiment was replicated three times with similar results. Data are represented as the mean ± SD (*n* = 3). ** *p* < 0.01 represents significant differences compared with the vehicle control (without LPS); ^#^ *p*<0.05; ^##^ *p* < 0.01 represent significant differences compared with the LPS-treated vehicle.

Excess production of prostaglandin E_2_ (PGE_2_) is closely linked to inflammation, fever, pain, and cancer [37]. To examine whether SM or PM inhibits PGE_2_ release in activated macrophages, culture supernatants were analyzed using commercial ELISA kits. Figure 6a shows that LPS (100 ng/mL) treatment for 18 h significantly induced PGE_2_ production from the basal level of 14.1 ± 4.7 to 46.8 ± 5.8 ng/mL, and co-treatment with PMB (10 μg/mL) reduced PGE_2_ production to 27.7 ± 1.8 ng/mL. SM (0.05 and 0.1 mg/mL) almost completely abolished PGE_2_ production to the basal level. On the other hand, a high concentration of PM (0.1 mg/mL) slightly inhibited LPS-stimulated PGE_2_ production to 35.1 ± 3.1 ng/mL (*p* < 0.05), but a lower concentration of PM (0.05 mg/mL) did not exert inhibitory effect.

Since PGE_2_ synthesis has been implied in up-regulation of COX-2, we investigated whether SM and PM prevent the LPS-induced expression of COX-2 protein using Western blot analysis. Figure 6b,c show that incubation of cells with LPS (100 ng/mL) for 16 h increased COX-2 protein expression by about 7.2-fold, as compared with the vehicle, and co-treatment with PMB (10 μg/mL) completely blocked the overexpression. However, SM (0.05 and 0.1 mg/mL) and PM (0.1 mg/mL) significantly enhanced LPS-mediated up-regulation of COX-2 expression. Similarly, COX-2 specific and non-specific inhibitors, such as NS-398 and ibuprofen, have also been reported to induce COX-2 protein and mRNA expression [38]. This result suggests that SM and PM may serve as COX enzyme activity inhibitors, instead of gene down-regulators.

#### 2.3.5. The Inhibitory Effects of Methanol Fractions of Seed Shell (SM) and Seed Pomace (PM) on LPS-Induced IL-6 Release and IL-1β mRNA Expression

**Figure 7:**
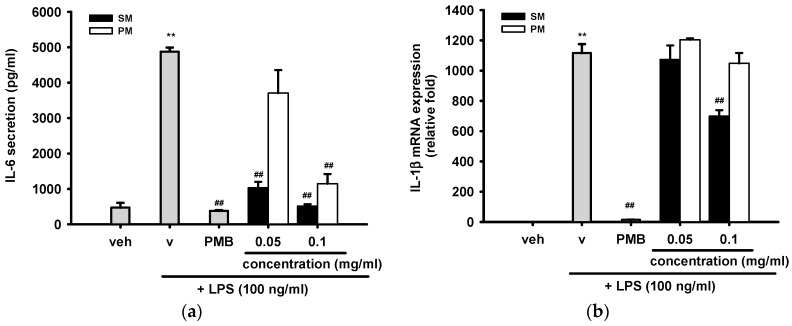
The inhibitory effects of methanol fractions of seed shell (SM) and seed pomace (PM) on LPS-induced IL-6 release and IL-1β mRNA expression. (**a**) RAW 264.7 cells were treated with indicated reagent for 18 h and the culture media were collected for IL-6 assay, as described in Materials and Methods; (**b**) RAW 264.7 macrophages were cultured with indicated reagent in six-well plates for 12 h. Total RNA was prepared and the mRNA levels of IL-1β were quantified by RT-Q-PCR relative to β-actin, as described in Materials and Methods. Data are represented as the mean ± SD (*n* = 3). ** *p* < 0.01 represents significant differences compared with the vehicle control (without LPS); ^##^ *p* < 0.01 represents significant differences compared with the LPS-treated vehicle.

Interleukin-6 (IL-6) is one of the major pro-inflammatory cytokines produced by monocytes and macrophages. Higher level of IL-6 is involved in autoimmune disorders and chronic inflammation. Figure 7a shows that treatment of RAW 264.7 cells with LPS (100 ng/mL) for 18 h strongly induced IL-6 secretion by ~10-fold, and the addition of PMB (10 μg/mL) completely blocked its induction. SM (0.05 and 0.1 mg/mL) reduced LPS-induced IL-6 production by 79% and 89%, respectively. PM (0.1 mg/mL) inhibited IL-6 release by 76% (*p* < 0.01), but a lower concentration of PM (0.05 mg/mL) did not exert inhibitory effect.

IL-1α and IL-1β play a central role in many autoinflammatory diseases. They are the founding members of the IL-1 family, whose function is to control pro-inflammatory reactions in response to tissue injury by pathogen-associated molecular patterns or the damage- or danger-associated molecular patterns released from damaged cells [39]. Their cellular RNA levels and gene transcription rates are regulated in parallel in response to LPS, but IL-1β mRNA has longer stability than IL-1α in RAW 264.7 cells [40]. Figure 7b demonstrates that LPS (100 ng/mL) treatment for 12 h caused a more than 1000-fold increase in IL-1β mRNA expression and PMB (10 μg/mL) almost completely attenuated its induction. On the other hand, only a high concentration of SM (0.1 mg/mL) could lower LPS-mediated IL-1β transcription by ~37%, and the other test samples had no detectable effect.

## 3. Discussion

Numerous studies have shown that many of the phytochemicals, especially polyphenols, are potent antioxidant, anti-inflammatory, anti-aging, anti-viral, and anti-bacterial agents, which may account for their health promoting properties [41,42]. To evaluate the antioxidant potency of the oil production byproducts from *Camellia tenuifloria*, the crude ethanol extracts and different partition fractions of fruit shell, seed shell, and seed pomace were analyzed for their scavenging effects against DPPH free radicals and total phenolic contents. Our results demonstrated that the crude ethanol extracts of fruit shell and seed shell (FE and SE) contained higher phenolic contents than that of seed pomace (PE) and exhibited stronger DPPH scavenging activities. In addition, methanol fractions (FM, SM, and PM) had the highest phenolic contents and antioxidant activities as compared with other partition fractions. These results indicated that the principal antioxidant components in the methanol fractions might be polyphenolic compounds. On the other hand, water-soluble non-phenolic compounds could be responsible for the DPPH scavenging activity of the aqueous fraction of seed shell (SA). It was reported that kaempferol glycosides are the major compounds responsible for the antioxidant activities in the seed pomace of *C. oleifera* and *C. tenuifloria* [15,43]. The DPPH scavenging activities of FM, SM, and PM were more potent than those reported for kaempferol glycosides, indicating other antioxidant compounds remain to be identified. In addition, it has been reported that different classes of phytochemicals have variable antioxidative strength and the additive and synergistic effects of phytochemicals may be responsible for their potent antioxidant [44].

Many plant extracts and isolated natural compounds have been reported to have anti-tyrosinase activities, with flavonoids identified as the main constituents of these [23]. Like most of the coupled enzyme reaction assays, a marked lag period was observed when the enzymatic reaction was started with l-tyrosine, characteristic of monophenolase activity [31,45]. The lag phase depends on the concentrations of enzyme and substrate. It can be shortened, or even abolished, by the presence of transition metal ions or *o*-diphenols [22]; but can be extended by some monophenolase inhibitors [46]. Using l-tyrosine and DOPA as substrates, we reported for the first time that the fruit shell and seed shell of *C. tenuifloria* exhibited both monophenolase and diphenolase inhibitory activities. The ethanol extract of the seed shell was more effective than that of fruit shell, but much weaker than kojic acid, whose action is attributed to chelating copper ion in the tyrosinase active site and exhibited a lag phase when l-tyrosine was used as a substrate. 

The tyrosinase inhibitors of the seed shell were distributed in different partition fractions, indicating the possible existence of heterogeneous chemicals responsible for the effects. On the hand, most of the tyrosinase inhibitory activity of fruit shell was concentrated in the *n-*butanol fraction. Several studies have reported that the number and position of hydroxyl group of the B ring of flavonoids and the substituents are important in the tyrosinase inhibitory activity [23]. Inhibitors of other classes, such as terpenes, steroids, chalcones, alkaloids, long-chain fatty acids, coumarins, sildenafil analogs, bipiperidines, biscoumarins, oxadiazole, tetraketones have also been reported [47]. Further isolation of active components, kinetic studies of tyrosinase inhibition and safety tests in cell culture are needed to assess the potential use of camellia oil byproducts as food additive or cosmetics. 

It has been reported that LPS cannot induce *iNOS* gene expression in human THP-1 monocytes and macrophages [48]. In addition, human peripheral monocytes and their derived macrophages are not able to express the *iNOS* gene after LPS induction [49,50,51]. Therefore, anti-nitric oxide activity in LPS-stimulated RAW 264.7 cells was regularly used as a screening platform for immunomodulatory agents used for human [52]. In the current study, we found that none of the crude ethanol extracts of the fruit shell, seed shell, and seed pomace from *C. tenuifloria* exhibited any NO inhibitory effect. However, the phenolic-rich methanol fractions of the seed shell and seed pomace (SM and PM) inhibited LPS-induced NO production without exerting cytotoxicity in RAW 264.7 cells. Western blot and RT-Q-PCR analyses further demonstrated that SM suppressed iNOS protein and mRNA expression in LPS-stimulated macrophages significantly (*p* < 0.01), indicating that SM inhibited NO generation in LPS-activated cells principally through repression of iNOS expression. However, the high concentration of PM (0.1 mg/mL) only slightly repressed iNOS mRNA, but not protein expression, suggesting that PM might have more effect on iNOS enzyme activity than on gene expression. In comparison, fractions from fruit shell, such as *n-*hexane fraction (FH), phenolic-rich methanol fraction (FM) and *n-*butanol fraction (FB) significantly enhanced LPS-induced cytotoxicity so as to inhibit nitric oxide production. 

HO-1 expression induced by stress provides protective effects, and it has been proposed that HO-1 induced by LPS allows the macrophages to survive from injuries initiated by the LPS-driven oxidative burst [53]. Various reports also support the view that HO-1 induction results in the down-regulation of inflammatory responses [34,35,54,55]. Current data reveal that both SM and PM enhanced LPS-mediated HO-1 mRNA and protein expression, and the former was more potent. HO-1 activity has also been shown to be involved in the anti-inflammatory action of SM, but not PM, because the addition of ZnPP, the competitive inhibitor, partially reversed ME’s inhibitory activity against LPS-stimulated NO release. On the other hand, PMB blocks HO-1 induction and inflammatory gene expression in response to LPS, because it neutralizes the endotoxin by directly binding to lipid A. 

PGE_2_ is produced from arachidonic acid to PGH_2_ via COX-1 and COX-2 activity. PGH_2_ is then converted to PGE_2_ by microsomal PGE synthase-1 (mPGES-1) [30]. In this research, we found that SM (0.05 and 0.1 mg/mL) strongly inhibited PGE_2_ production, supporting its potent anti-inflammatory activity. However, it significantly enhanced, rather than attenuated, LPS-mediated COX-2 protein expression. Weaker inhibition of PGE_2_ production and COX-2 up-regulation was also observed for PM (0.1 mg/mL). We continued to investigate how SM and PM affected LPS-mediated COX-2 and mPGES-1 transcription. Supplemental Figure S1 shows that both SM and PM (0.05 and 0.1 mg/mL) caused significant additive up-regulation of the mRNA expression of COX-2 and mPGES-1 in response to LPS. COX-2 specific and non-specific inhibitors have also been reported to induce COX-2 protein and mRNA expression [38,56]. It is therefore possible that SM and PM served as COX and/or mPGES-1 enzyme inhibitors so as to decrease PGE_2_ production. Aside from COX-dependent synthesis, PGE_2_ levels are also regulated by its degradation mediated via two principal PGE_2_-inactivating enzymes, 15-hydroxy-PG dehydrogenase (15-PGDH) and carbonyl reductase (CR) [57]. Further investigation is needed to better clarify the underlying PGE_2_ diminishing mechanisms of SM and PM.

We found that in parallel to effects on LPS-mediated NO and PGE_2_, SM inhibited IL-6 production more potently than PM. SM (0.1 mg/mL) also slightly down-regulated IL-1β expression. It has been reported that transcription factors NF-IL6 and NF-κB synergistically activate expression of IL-6 and iNOS in response to LPS in macrophages [58,59]. Our results indicate that neither SM nor PM were able to attenuate LPS-mediated NF-κB activation (data not shown). However, the exact transcription factor affected by SM and PM remains unknown at this stage. 

## 4. Materials and Methods

### 4.1. Materials

Ripe fruits of *C. tenuifloria* were collected from farmers in Ruisui Township, Hualien County in Nov. 2014. DPPH (2,2-Diphenyl-1-(2,4,6-trinitrophenyl)hydrazyl), Folin-Ciocalteu reagent, sodium carbonate, gallic acid, mushroom tyrosinase, tyrosine, 3,4-dihydroxyphenylalanine (l-DOPA), Griess reagent, MTT (3-(4,5-dimethylthiazol-2-yl)-2,5-diphenyl tetrazolium bromide), lipopolysaccharide (LPS) from *Escherichia coli* O111:B4, polymyxin B sulfate (PMB), sodium nitrite, zinc protoporphyrin IX (ZnPP) and other chemicals were purchased from Sigma-Aldrich Co. (St. Louis, MO, USA), unless otherwise indicated.

### 4.2. Cold Percolation Extraction and Partition of Fruit Shell, Seed Shell, and Seed Pomace of C. tenuifloria

The ripe fruits of *C. tenuifloria* were collected and sun dried for about 1 week until they cracked. The fruit shells and seeds were separated and further sun dried until completely dry. The seed shell was then separated from seed kernel before roasting. The kernel was roasted at about 85 °C and then pressed with a screw oil press. The fruit shell and seed shell were dried in an oven at 50 °C to a constant weight and then powdered. The seed pomace was cut into small pieces, dried at 50 °C, powdered and reflux with *n*-hexane in a Soxhlet extractor. The dry sample (1 kg) was soaked in a small amount of 95% ethanol and then packed in a column and extracted with a sufficient amount of 95% ethanol at room temperature until the color of the eluent was clear and the amount of ethanol used was about four liters. The ethanol extracts were concentrated *in vacuo* to yield dark-brown syrup of 76.4, 79.8, and 90.9 g for fruit shell, seed shell and seed pomace, respectively. Small portions of the resins were dissolved in ethanol and denoted as FE, SE, and PE for the ethanol extracts of the fruit shell, seed shell and seed pomace, respectively.

Ten grams of the brown resin obtained above was re-suspended in H_2_O (50 mL) and partitioned with two volumes of ethyl acetate (EA) three times to yield the EA and the aqueous layers. The combined EA layer was concentrated in a rotary evaporator at 55 °C, and the resulting residue was dissolved in methanol and partitioned with an equal volume of *n*-hexane three times to yield the methanol and *n*-hexane fractions. 

**Figure 8:**
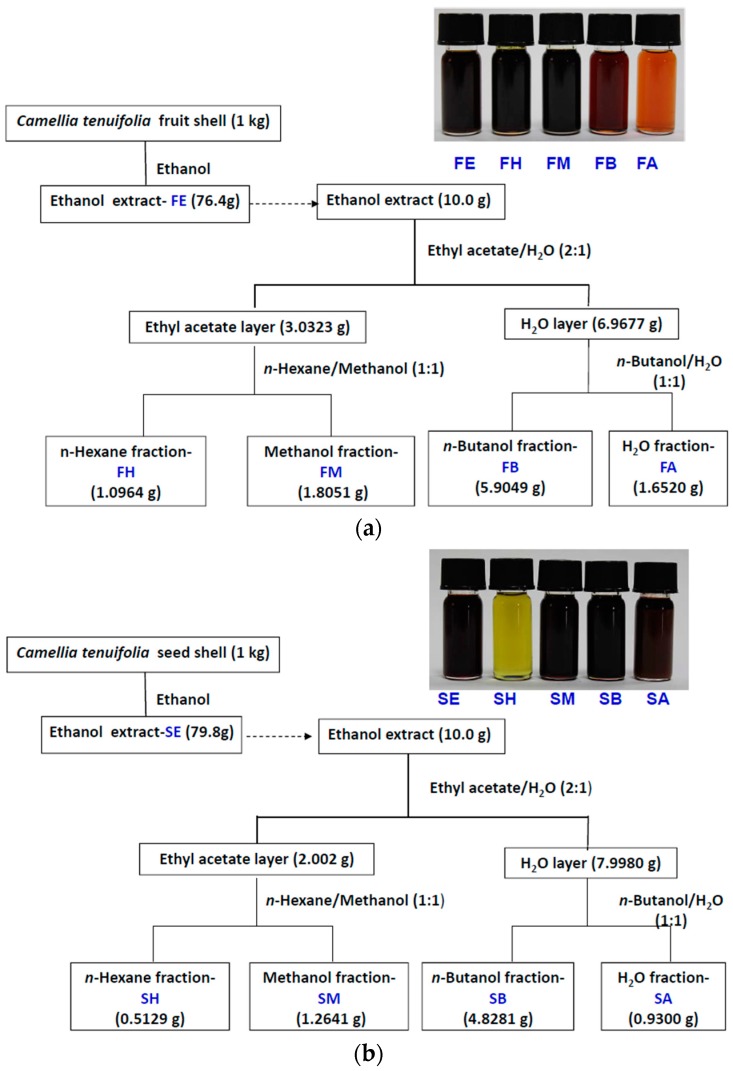

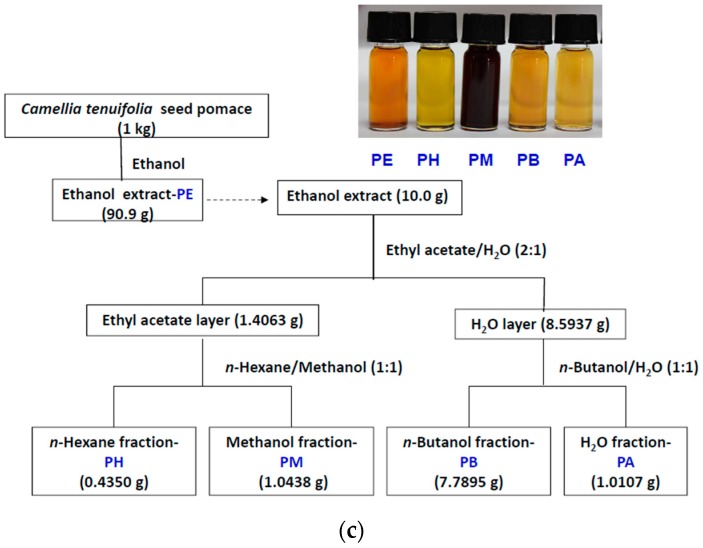
Scheme for cold percolation extraction and partition of the (**a**) fruit shell; (**b**) seed shell; and (**c**) seed pomace of *C. tenuifloria*.

The rest of the aqueous layer was further partitioned with an equal volume of *n*-butanol three times to give the *n*-butanol and aqueous layers. The combined *n*-butanol layer was concentrated in a rotary evaporator at 70 °C, and the resulting brown resin was dissolved in DMSO and denoted as the *n*-butanol fraction. The aqueous layer was lyophilized, and the resulting powder was dissolved in water and denoted as the aqueous fraction. Figure 8a–c shows the flow charts and yields of the extraction and partition for fruit shell, seed shell, and seed pomace of *C. tenuifloria*, respectively. 

### 4.3. DPPH Scavenging Capacities

The test samples were evaluated for their abilities to scavenge the stable DPPH radical (0.1 mM in methanol) according to the method presented in an earlier work [60]. The ability of the test material to quench the DPPH free radical was evaluated according to the following equation: scavenging % = (Ac − As)/Ac × 100%. As and Ac are the absorbances at 517 nm of the reaction mixture with sample and control, respectively. 

### 4.4. Folin–Ciocalteu Assay

Total phenolic content was determined by the slightly modified Folin-Ciocalteu (F-C) assay [61]. Eight μL of F-C reagent and 20 μL of the proper dilution of the test sample were added to each well of a 96-well plate, mix and stand for 10 min. Two hundreds μL of 2% aqueous sodium carbonate solution was added, mixed, and incubated at room temperature for 10 min. Absorbance was read at 620 nm and the results are expressed as minigrams of gallic acid equivalent per gram of dry weight (mg·GAE/g·dw).

### 4.5. Mushroom Tyrosinase Inhibition Assay and IC_50_ Determination

The monophenolase and diphenolase activity assays were performed with the modifications reported in Sato and Toriyama [46]. For monophenolase activity assay, 350 μL of 1 mM l-tyrosine was mixed with 245 μL of 50 mM phosphate buffer. Seven μL of the tested solution at the concentrations needed or vehicle (control) was then added. Finally, 105 μL of mushroom tyrosinase (200 U/mL in 50 mM phosphate buffer, pH 6.5) was added and mixed. On the other hand, for diphenolase activity assay, 350 μL of 1.5 mM 3,4-dihydroxyphenylalanine (l-DOPA) was mixed with 315 μL of 50 mM phosphate buffer. Seven μL of the tested solution at the concentrations needed or vehicle (control) was then added. Finally, 35 μL of mushroom tyrosinase (200 U/mL in 50 mM phosphate buffer, pH 6.5) was added and mixed.

The optical density of the sample at 490 nm was monitored at 26 ± 0.5 °C by a double beam spectrophotometer (Hitachi U2800) for 15 min relative to the reference (without tyrosinase). To calculate the initial velocity of tyrosinase activity (ΔA_490_/min), the slope of the early linear range of the enzyme reaction progress plot was made using the observed absorbance *vs.* time. The fractional activity (*Vi*/*Vo*) was determined as the ratio between the initial velocity in the presence (*Vi*) and absence (*Vo*) of the inhibitor. The IC_50_ values were calculated from the regression analysis of fractional activity *vs.* concentration plot, and denoted the concentration of the sample required to inhibit 50% of the enzyme activity. Kojic acid, dissolved in 50 mM phosphate buffer, was used as a positive control. Each measurement was made at least in triplicate. 

### 4.6. Culture and Measurement of Nitrite, Prostaglandin E_2_ (PGE_2_) and IL-6 Release in RAW 264.7 Cells

RAW 264.7 cells were purchased from the Bioresource Collection and Research Center (Hsinchu, Taiwan) and cultured in DMEM with 10% fetal bovine serum (HyClone, Logan, UT, USA), 2 mM glutamine, 1% non-essential amino acid, and 1 mM pyruvate (Invitrogen Life Technologies, Carlsbad, CA, USA). Cells were cultured at 37 °C in a humidified atmosphere of 5% CO_2_ and 95% air.

RAW 264.7 cells (1 × 10^6^/mL) in a 96-well plate were incubated with antibiotic polymyxin B (PMB, 10 μg/mL) or test sample in the presence of LPS (100 ng/mL) for 18–24 h. PGE_2_ and IL-6 in the supernatant was measured by Prostaglandin E_2_ ELISA Kit (Cayman Chemical, Ann Arber, MI, USA) and mouse IL-6 ELISA Set (BD Biosciences, San Diego, CA, USA), respectively. Equal volume of Griess reagent (1% sulphanilamid and 0.1% naphthylenediamine in 5% phosphoric acid) was added to the supernatant to measure nitrite production. Absorbance was read at 550 nm and calculated against a sodium nitrite standard curve.

### 4.7. Cell Viability

Cell viability was assessed by the mitochondrial-dependent reduction of 3-(4, 5-dimethylthiazol-2-yl)-2, 5-diphenyl tetrazolium bromide (MTT) to purple formazan [62]. 

### 4.8. Western Blotting Analysis

Confluent RAW 264.7 cells (1 × 10^6^/mL) were incubated with vehicle, LPS plus vehicle, or LPS plus test sample (0.05 and 0.1 mg/mL). After being incubated for 16 h, cell lysate was prepared using RIPA buffer (Thermo Fisher Scientific, Inc., Rockford, IL, USA) and the protein concentration was determined by the Bradford method (Bio-Rad Laboratories, Hercules, CA, USA) using bovine serum albumin as a standard. 

Equal amounts of cell lysates were separated on SDS-PAGE (8%–12%) and then transferred onto Hybond-P PVDF (GE Healthcare, Buckinghamshire, UK) using a CAPS transfer buffer at 20 volt overnight at 4 °C. The membranes were blocked at room temperature in a freshly made blocking buffer (5% skim milk in PBS with 0.05% Tween 20, pH 7.4) for 6 h. The membranes were then incubated overnight at 4 °C in blocking buffer containing appropriate dilution (1:1000–1:5000) of primary antibody (Table 3). The membranes were then incubated for 1 h at room temperature with suitable horseradish peroxidase-conjugated secondary antibody (Jackson ImmunoResearch, West Grove, PA, USA) at a dilution of 1:10,000–1:25,000. The proteins of interest were detected by ECL Prime (GE Healthcare) and the chemiluminescent signals were then visualized with X-ray film. Densitometry of the bands was analyzed by ImageJ software (National Institutes of Health, Bethesda, MD, USA). 

**Table 3:** Primary antibodies used in Western blotting.

Antibody	Company	Catalog Number
α-tubulin	Sigma	T6199
NOS	Cell Signaling	2977
COX-2	Santa Cruz	Sc-166475
HO-1	Stressgen	SPA-895

### 4.9. RNA Extraction and Reverse Transcription Real-Time PCR 

Total cellular RNA was extracted from RAW 264.7 cells using Illustra RNAspin Mini RNA Isolation Kit (GE Healthcare). An aliquot of 0.8 μg RNA was used to synthesize cDNA using a High-Capacity cDNA Archive kit (Applied Biosystems, Foster City, CA, USA). Quantitative PCR was performed with 2 μL of the cDNA obtained above in a 20 μL solution containing 200 nM primers (Table 4) and Power SYBR^®^ Green PCR Master Mix (Life Technologies). Amplification was conducted in an ABI StepOne Real Time PCR System. PCR conditions were as follows: 95 °C for 2 min, 40 cycles at 94 °C for 15 s, and 60 °C for 60 s. The cycle threshold (*C*_t_) values of each gene and the internal control β-actin were obtained and the relative quantification for each gene was calculated using the ΔΔ*C*_t_ method.

**Table 4:** Primer pairs used in RT-Q-PCR.

Gene	Primers	Amplicon (bp)
β-actin	GGCTGTATTCCCCTCCATCG, CCAGTTGGTAACAATGCCATGT	154
iNOS	GTTCTCAGCCCAACAATACAAGA, GTGGACGGGTCGATGTCAC	127
HO-1	AAGCCGAGAATGCTGAGTTCA, GCCGTGTAGATATGGTACAAGGA	100
IL-1β	TTCAGGCAGGCAGTATCACTC, GAAGGTCCACGGGAAAGACAC	75
COX-2	TGAGCAACTATTCCAAACCAGC, GCACGTAGTCTTCGATCACTATC	74
mPGES-1	ATGAGGCTGCGGAAGAAGG, GCCGAGGAAGAGGAAAGGATAG	150

### 4.10. Statistical Analysis

All experiments were repeated at least three times. The results were presented as means ± SD and analyzed by the Kruskal–Wallis Test. A *p* value of <0.05 was taken to be significant. If the Kruskal-Wallis Test shows a significant difference between the groups, then pairwise comparisons were used by employing the Mann–Whitney *U* Tests.

## 5. Conclusions

This research shows for the first time the possible applications of oil production residues from *Camellia tenuifloria* in the food additive, medicine, and cosmetic industries. The crude ethanol extracts of fruit shell and seed shell (FE and SE) contained more phenolic contents than that of seed pomace (PE), and exhibited stronger antioxidant and anti-tyrosinase activities. Among all of the partition fractions, the methanol and aqueous fractions of seed shell (SM and SA) exhibited the strongest DPPH scavenging activities, indicating both phenolic and non-phenolic antioxidants existing in the seed shell. In contrast, the strongest anti-tyrosinase activities were in the butanol and aqueous fractions of the seed shell (SB and SA). The methanol fractions of seed shell (SM) and seed pomace (PM) were the only fractions which exhibited anti-inflammatory activity without exerting cytotoxicity in RAW 264.7 cells, and the activity of SM was much stronger than that of PM. SM reduced NO production, iNOS protein and mRNA expression in LPS-activated RAW 264.7 cells. It also repressed the expression of IL-1β, and the production of PGE_2_ and IL-6 in response to LPS. The up-regulation of HO-1 expression contributed, at least in part, to the anti-inflammatory action of SM. In conclusion, the seed shell of *C. tenuifloria* is a potent antioxidant, anti-tyrosinase and anti-inflammatory agent, thereby warranting further investigations into its active components and underlying mechanisms.

## Supplementary Materials

Supplementary materials can be found at http://www.mdpi.com/1422-0067/16/12/26184/s1.
