# Supplementary material for: Antioxidant, Anti-Tyrosinase and Anti-Inflammatory Activities of Oil Production Residues from Camellia tenuifloria
Source: Int J Mol Sci. 2015 Dec 10;16(12):29522–41. doi: 10.3390/ijms161226184 (PMC4691127; doi:10.3390/ijms161226184)
Supplement: Supplementary file 1 [file ijms-16-26184-s001.pdf]

Supplementary Materials: Antioxidant, Anti-Tyrosinase, and Anti-inflammatory Activities of Oil Production Residues from *Camellia tenuifloria*

Shu-Yuan Chiou, Choi-Lan Ha, Pei-Shan Wu, Chiu-Ling Yeh, Ying-Shan Su, Man-Po Li and Ming-Jiuan Wu

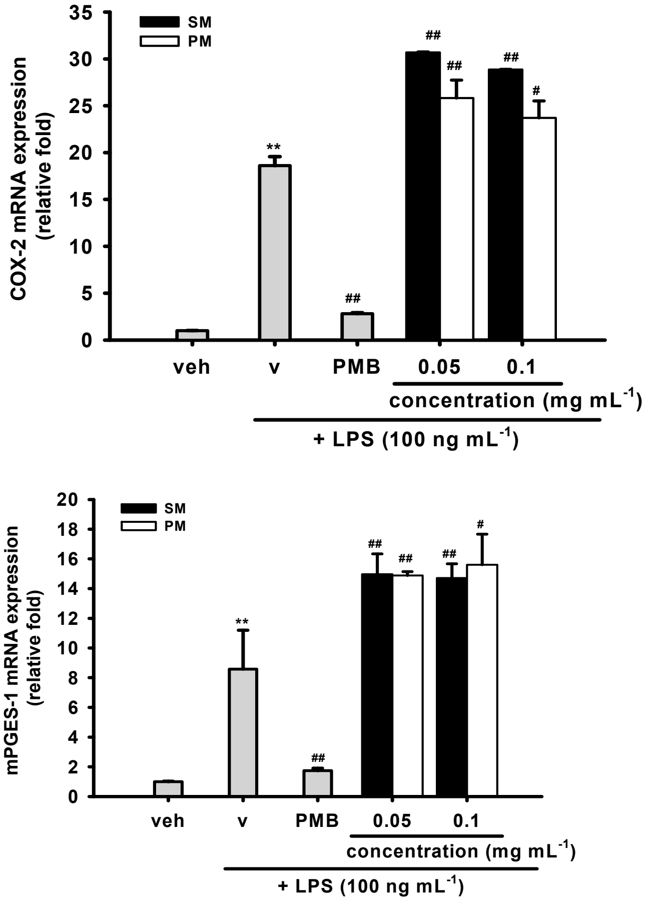

Figure S1. Effects of methanol fractions of seed shell and seed pomace on LPS-induced mRNA expression of COX-2 and mPGES-1. RAW 264.7 cells were treated with either vehicle (0.1% DMSO), LPS (100 ng/mL) in the presence of vehicle, polymyxin B (PMB, 10 μg/mL), methanol fraction of seed shell (SM), or methanol fraction of seed pomace (PM) at the indicated concentrations for 12 h. Total RNA was prepared and the mRNA levels of COX-2 and mPGES-1 were quantified by RT-Q-PCR relative to β-actin, as described in Materials and Methods. Data represent the mean ± SD of three independent experiments. ** $p < 0.01$ represents significant differences compared with the vehicle control (without LPS). # $p < 0.05$; ## $p < 0.01$ represent significant differences compared with the LPS-treated vehicle.
